# 

**Published:** 1877-03-20

**Authors:** 


### CONTENTS.

ORIGINAL AND SELECTED ARTICLES—

A Recent Treatment for Irreducible Retroflexion of the Uterus. By A. H. Goelet, M.D. ... 57
Tracheotomy for the Removal of a Foreign body. By R. Inge, M. D. ... 58
Ammonia in Bronchial and Lung Troubles. A. H. Patton, M.D. ... 59
Cases Bearing on Some Doubtful Points in the History of Syphilis. By Osgood Mason, M.D. ... 60
Gelseminum Sempervirens ... 62
Delayed Labor and Death of Patient ... 64
Chronic Dysentery Topically Treated. By T. Wertz, M.D ... 65
A Case of Diabetes Mellitus Treated with Chloral Hydrate—Relieved. By E. Cross, M.D ... 67
Bronchitis ... 68

ABSTRACTS AND GLEANINGS—
Stimluants in Typhoid Fever, 69. Dilatation of the Uterus, 70. Treatment of "Chronic Diarrhæ" by Koumiss, 71. The Iodides in Lead-Poisoning, 71. Impotence, 72. Chronic Eczema, 72. Hot Water In Croup, 72. On the Treatment of Ranula, 73. Protagon, 73. Tic Doloreux of the Face Cured by Bromide of Potassium, 73. To Check Colliquative Sweating, 74. Raw Onion as a Diuretic, 74. The Relief of Prickly Heat, 74. New Anæsthetic Agent, 75. Antihydropin, 75. A Combination of Tæniafages, 75. Hay Fever, 75. Ergot in Typhoid Fever, 76. Jaborandi as a Galactagogue, 76. Subcutaneous injections of Cold Water in Acute Rheumatism, 76. An Emmenagogue Pill, 76. Carbolic Spray in Bronchial Catarrh, 76 Junctus Acutus in Ascites, 76. Laxative Antibilious Pills, 77. Potion, 77. Fetid Feet, 77. Chromic Acid for Warts, 77. Treatment of Acute Articular Rheumatism by Cyanide of Zinc, 77. Formula for Diphtheria, 77. The Cold Douche in the Treatment of Buboes, 77. Colored Spectacles, 77. Antidotes of Arsenic, 78. Salicylate of Soda in Rheumatism, 78. Nitric Acid for Hoarseness, 78. Hot packing in Rheumatism, 78. Safrol, 78. Chloral Plaster, 78. Tinea Tonsurans, 81. Small Poisonoue Doses of Chloral in Delirium Tremens, 81. Therapeutic effects of Corrosive Sublimate in Blummorrhœa, 81. Arsenic in the Stomach, 82. Bromide of Potassium in Hæmorrhage, 82.

PRESCRIPTIONS AND FORMULÆ—Strumous Opthalmia, Improved Cathartic Pills, For Asthmatic Paroxysm, LaFayette Mixture, Emulsion of Phosphorus, Alterative and Tonic for Chronic Pharyngitis, For Asthma in Intervals, 79.

PRACTICAL HINTS—80.

SCIENTIFIC ITEMS—82.

EDITORIAL AND MISCELLANEOUS—
Our Colaborers, Our Size, The United States Pharmacopea, Transactions of the Medical Society of Virginia, American Microscopical Society, 83. Transactions of the Mississippi State Medical Association, 84.

BOOK REVIEWS—84.

